# An Assessment of the Altimetric Information Derived from Spaceborne SAR (RADARSAT-1, SRTM3) and Optical (ASTER) Data for Cartographic Application in the Amazon Region

**DOI:** 10.3390/s8063819

**Published:** 2008-06-06

**Authors:** Cleber Gonzales de Oliveira, Waldir Renato Paradella

**Affiliations:** National Institute for Space Research (INPE), Remote Sensing Division (DSR). São José dos Campos, 12227- 010. São Paulo State, Brazil; E-mails: cleber@dsr.inpe.br; waldir@ltid.inpe.br

**Keywords:** SRTM3, RADARSAT-1, ASTER, topographic mapping, Amazon Region

## Abstract

Difficulties in acquiring a complete aerial photography coverage on a regular basis in the Brazilian Amazon due to adverse environmental conditions affect the quality of the national topographic database. As a consequence, topographic information is still poor, and when available needs to be up-dated or re-mapped. In this research, altimetric information derived from RADARSAT-1 (Fine and Standard modes), SRTM3 (3 arc-seconds) and ASTER (band 3N-3B) was evaluated for topographic mapping in two sites located in the region: Serra dos Carajás (mountainous relief) and Tapajós National Forest (flat terrain). The quality of the information produced from Digital Elevation Models (DEMs) was evaluated regarding field altimetric measurements. Precise topographic field information acquired from Differential Global Positioning System (DGPS) was used as Ground Control Points (GCPs) for the modeling of the stereoscopic DEMs (RADARSAT-1, ASTER) and as Independent Check Points (ICPs) for the calculation of accuracies of the products. The accuracies were estimated by comparison of the DEMs values and real elevation values given by ICPs. The analysis was performed following two approaches: (1) the use of Root Mean Square Error (RMSE) for the overall classification of the DEMs considering the Brazilian Map Accuracy Standards (PEC) limits and, (2) calculations of trend analysis and accuracy based on a methodology that takes into account computed discrepancies and standard deviations. The investigation has shown that for flat relief, the altimetric accuracy of SRTM3 and Fine RADARSAT-1 DEMs fulfilled the PEC requirements for 1:100,000 A Class Map. However, for mountainous terrain, only the altimetry of SRTM3 and ASTER fulfilled these requirements. In addition, the performance of ASTER was slightly superior to SRTM3. However it is important to consider the difficulties in the acquisition of good stereo-pairs with optical data in the Amazon and the additional cost (GCPs) to produce ASTER DEMs. Despite showing systematic errors, the findings justify the usage of SRTM3 as a primary elevation source for semi-detailed topographic mapping in the region. It is suggested a combination of altimetry derived for SRTM3 and planimetry extracted from high-resolution SAR (ALOS/PALSAR, TerraSAR-X, RADARSAT-2) or if available optical data for semi-detailed topographic mapping programs in the Brazilian Amazon, where terrain information is seldom available or presents low quality.

## 1. Introduction

Topographic mapping in large areas of the Brazilian Amazon region has always been a challenge, even under the most suitable field infrastructure. Due to the adverse environmental condition (perennial cloud cover, rain, smoke during dry season, dense rainforest, poor access), the usage of optical remote sensing (RS) data for regular basis coverage is expensive or even not possible. As a consequence, the topographic knowledge is still poorly known, with almost 25% of the area covered by maps at reconnaissance scale (1: 250,000 up to 1:1,000,000). For the remainder of the region, the cartography at semi-detailed scale (1:100,000) needs to be up-dated or re-mapped [1]. The integration of the region to the economy of the remainder of the country is in a fast pace for the last four decades driven by a road network established in the sixties and by internal migration of agricultural and mining workers in search for better opportunities. Such changes in the demographic and land use patterns require thorough planning by State and federal levels administrations. Government infrastructure planning, resources assessment, monitoring and management are based on geospatial information. The primary inputs supporting all geographic information are topographic maps.

Digital Elevation Model (DEM) is a primary input for topographic mapping. With the launch of the Canadian RADARSAT-1 in 1995, DEMs could be for the first time systematic generated using orbital SAR stereoscopy (or radargrammetry). On the other hand, with the ASTER sensor, launched in 1999 on board of Terra platform, it was also possible the generation of optical stereoscopic DEMs from the VNIR band 3 (nadir and backwards). Results have been published in the literature with a general consensus of the elevation accuracy with RADARSAT-1: around 20 m for Standard and 12 m for Fine stereo-pairs [2]. With ASTER stereo-pairs elevation accuracy has been published ranging from 7 m to 18 m [3, 4]. However, the extrapolation of these findings for operational use in the moist tropics should be taken with caution. Firstly, these results were obtained in sites with favorable environmental (low to moderate vegetation cover, well defined ground features within the RS images, etc.) and experimental conditions (ancillary planialtimetric data with high quality and quantity to derive DEMs and test the accuracies). Secondly, ground features are poorly expressed in RS images in tropical environment, particularly when dealing with SAR. Thus, GCPs with quality, number and distribution is a critical point for the operational use of the technology in the moist tropics.

With the SRTM mission in February 2000, interferometric DEMs were also available for the globe [5]. Based on [6] the performance for DEMs derived from SRTM data sampled over a grid of 1 arc-second by 1 arc-second (30 m by 30 m) for South America showed an absolute geolocation and height errors (90 % of probability) of 9 and 6.2 meters, respectively. On the other hand, results related to the use of altimetry produced from SRTM3 (3-arc seconds by 3-arc seconds) in the tropics (Ecuador, Honduras, Colombia) indicated an average error of 8 m in elevation and overall quality more accurate than available on 1: 50,000 scale cartographically derived air-photos DEM [7]. In the present paper, the altimetric quality of DEMs generated from two sources of SAR (RADARSAT-1, SRTM3) and optical (ASTER) data was evaluated for two distinct terrains in the Brazilian Amazon. This paper is an outgrowth of previous radargrammetric investigations in the Brazilian Amazon [8]. It shows a practical example of how the altimetry derived from orbital SAR can be applied to overcome the critical lack of topographic information in the Amazon Region.

## 2. Test-sites

**Figure 1.**
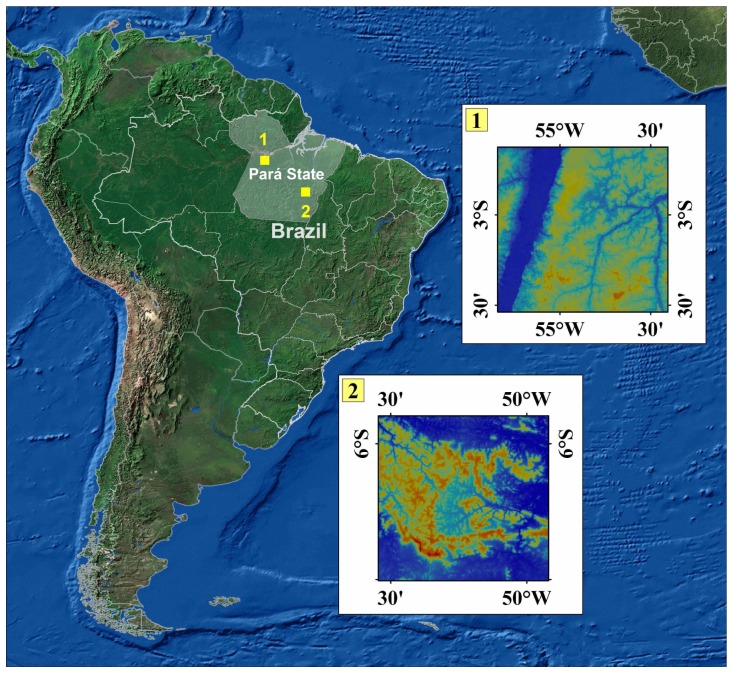
Location map of the study areas (1 = Tapajós, 2 = Carajás).

The investigation was carried out in two test-sites located in the central part and in the easternmost border of the Brazilian Amazon region, respectively (Figure 1). The first area was selected at the lower Tapajós River region, within the Belterra municipality. It is characterized by low terrain (altitudes from 30 to 170 meters). Geologically, the area is within the Amazonas Sedimentary Basin, with fluvial to lacustrine sediments of the Alter do Chão Formation, part of the Javari Group, with Tertiary age. The vegetation is also typical of the dense tropical forest showing high plateaus with emergent trees and uniform cover (Dense Ombrophilous Forests of Lowlands), and sections of low and dissected plateaus with few emergent and high density of palm trees (Open Ombrophilous Forests). Land use is related to subsistence agriculture, few cash crops and cattle raising. The site encompasses around 1,940 km^2^ and comprises part of two 1:100,000 scale topographic sheets produced from black and white air photos by Brazilian Army (DSG) during the 1973-1983 period.

The second area is within the Carajás Province, the most important Brazilian mineral province with the world's largest iron deposits. The region is characterized by a set of hills and plateaus known as Serra dos Carajás (altitudes from 500 to 900 meters) surrounded by southern and northern lowlands (altitudes around 200 meters), totally covered by Ombrophilous Equatorial forest. Geologically the Province includes basement and volcano-sedimentary rocks of the Archean Itacaiunas Shear Belt and anorogenic granites with Proterozoic ages. The study area with around 3,736 km^2^ encompasses the Água Azul do Norte, Canaã dos Carajás, Marabá and Parauapebas municipalities. It comprises the Serra dos Carajás topographic sheet (1:100,000 scale) produced from black and white air photos by the Brazilian Institute of Geography and Statistics (IBGE) during the 1979-1981 period.

## 3. Characteristics of the data set

The selection of the best radar stereo-pair is a function of sensor (intersection angle, proportion of overlap between beam positions, spatial resolution) and terrain parameters (expected elevation accuracy, size of the study area, elevation and slope, surface characteristics). Considering the RADARSAT-1 tracks over Carajás, three same-side stereo configurations were available for the investigation: two Fine mode pairs (F2/F5, ascending and descending passes) and one Standard mode pair (S5/S7, descending pass). For Tapajós area, only one descending F2/F5 pair was available. Results from literature [9] indicated that RADARSAT-1 stereo pairs with 6-8 degrees of intersection angle are enough to provide accurate elevation. This requirement was fulfilled with the chosen pairs in the research (6 degrees for Fine and 8 degrees for Standard pairs). Finally, in order to minimize radiometric variations induced by environmental changes at the terrain, the scenes of the pairs were acquired as close as possible: with the exception of the Standard pair. The ASTER stereo-pair for Carajás was acquired during the dry season. The main characteristics of the RADARSAT-1 and ASTER pairs are presented in Tables 1, 2, and 3.

## 4. Methodology

### 4.1. Generation of stereoscopic DEM

Two kinds of stereoscopy were addressed in the research: (1) the across-track stereoscopic using two distinct orbits (RADARSAT-1), and (2) the along-track stereoscopy with scenes from the same orbit using fore and aft viewing (ASTER). For the first approach, the RADARSAT-1 Specific model based on OrthoEngine package from PCI software [10] was used to compute the stereo model geometry and the 3D intersections for the DEMs generations. This model takes into account the satellite positioning information, reducing the requirement for numerous well-distributed ground control points for the DEM generation while maintaining positional accuracy and high levels of detail. It operates on a monoscopic basis, which considers each scene separately to form the model. The elevation is estimated by determining the solution, which satisfies the geometry, defined by the two (stereo) satellite positions, calculated slant ranges and Doppler planes. The addition of GCPs refines the model and improves its accuracy. Once the geometric model is computed, quasi-epipolar curve images are generated and the elevation parallax is derived based on automated image matching procedure, which utilizes a multiscale area correlation with a mean normalized cross-correlation approach [11]. It is important to mention that the correlation process using SAR images is affected by speckle. The use of speckle filtering has been addressed in literature with distinct results, but in order to improve the correlation Enhanced Frost speckle filtering (5 × 5 window) was used. After computing the elevation parallax and producing the DEM, post-processing procedures were necessary (removing blunders, filling the mismatched areas, smoothing and filtering). For the transformation of geometric into orthometric altitudes, the MAPGEO98 model was used [12]. It presents an absolute accuracy (related to the model) of 1.5 m and a relative accuracy of 1.0 cm/km (the error that takes into account the distance from the reference level).

### 4.2. DGPS Field Measurements

Although the requirements for the GCPs number are not specified for optical or SAR images, a larger number is normally recommended to improve accuracy for DEM extraction, with samples ideally chosen on a variety of locations and ground elevations, at the lowest and highest elevation. On the other hand, ICPs also play a key role in quality control for mapping production. Generally, a balance has to be reached between few ICPs, giving invalid accuracy estimation and an excessive number, providing a safe analysis but with unrealistic cost of acquisition in the field. According to [13] a minimum of 20 well-distributed ICPs is necessary within a map. It is important to mention that topographic maps in Brazil should be classified according to the National Map Accuracy Standards (PEC in Portuguese), defined by the decree 89,817 of 1984, which classifies map products in relation to geometric quality. PEC is a statistical indicator (90% of probability) for planialtimetric accuracy, corresponding to 1.6449 times the Standard Error (PEC = 1.6449 × SE), and considering equivalent the expressions Standard Error, Standard Deviation Error and RMSE. For a 1:100,000 scale A Class map, the SE corresponds to 16.66 m (1/3 of the equidistance of contour lines). Precise planialtimetric measurements from Differential Global Positioning System (DGPS) were acquired in both test-sites, and used as GCPs for the modeling of the DEMs and as ICPs for the calculation of altimetric accuracies. Two dual frequency receptors were used in the field for static DGPS measurements using vehicles and helicopters. A total of 47 static points were collected in Tapajós and 50 points in Carajás. In addition, around 35,000 kinematic measurements were also collected for both areas. The dataset was corrected for ionospheric and tropospheric effects. The maximum errors with a probability of 68.3% (1 σ) for the measurements in Tapajós were 6.52 cm (latitude), 19.66 cm (longitude) and 17.74 cm (geometric altitude). The maximum errors in Carajás were 18 cm (latitude), 75 cm (longitude) and 24 cm (geometric altitude).

### 4.3. SRTM DEMs

SRTM data were originally sampled on a 1×1 arc-second grid (SRTM1) and further averaged to create the 3×3 arc-second (90 m by 90 m) data set (SRTM3), used in this work and available in HGT file format, covering 1° × 1° on the ground. The data were acquired at ftp://e0srp01u.ecs.nasa.gov/srtm/version2/SRTM3 in Lat/Long coordinates with the WGS84 horizontal datum and the EGM96 vertical datum. The data were imported and the HGT files were merged into continuous DEM (PCI format), with 90 m pixel spacing, followed by the application of an interpolation function to fill no-data holes based on Geomatica Focus from PCI software.

### 4.4. Statistical Analysis

The accuracy and classification of the products were estimated by comparison of the DEMs elevation values and the real elevation given by ICPs. It is important to mention that ICPs related to areas with void-filled SRTM-3 data were not considered in the calculation. The analysis was performed following two approaches: (1) the use of RMSE (Root Mean Square Error) and LE90 (Linear Error with 90% Probability) for the overall classification of the DEMs considering the PEC limits and, (2) calculations of trend analysis and accuracy based on the methodology proposed by [14], which takes into account computed discrepancies and standard deviations. The aim of trend analysis is to check for the presence of systematic errors and it was based on the null hypothesis (H_0_ : Δ average = 0; H_1_: Δ average ≠ 0) whose acceptance or rejection is controlled by the computed Student tstatistics(**t**_|_**_x_**_|_) compared with the theoretical t_n-1;α_. Accuracy analysis uses comparison of the variance of sample deviations (Var (Δ_Z_)) to their respective pre-defined (tabled) values. The test is carried out using a hypothesis about the mean and standard deviation of the sample for each of the altimetric value. Chi-square (χ^2^) is the statistical procedure that was applied. The accuracy of the product can be estimated in the altimetric (Z) values using standard statistical methodology involving the comparison of a sample value of χ^2^_(A Class)_ for A class map and the tabled value of χ^2^_(n−1;α)_. The calculus of χ^2^_(A Class)_ is given by (n − 1) × (SD^2^ / SE^2^), where n is the number of ICPs, SD is the standard deviation and SE corresponds to 16.66 m (1/3 of the equidistance of contour lines) for a 1:100,000 scale A Class map, as presented before.

### 4.5. Discussion of the results

**Figure 2.**
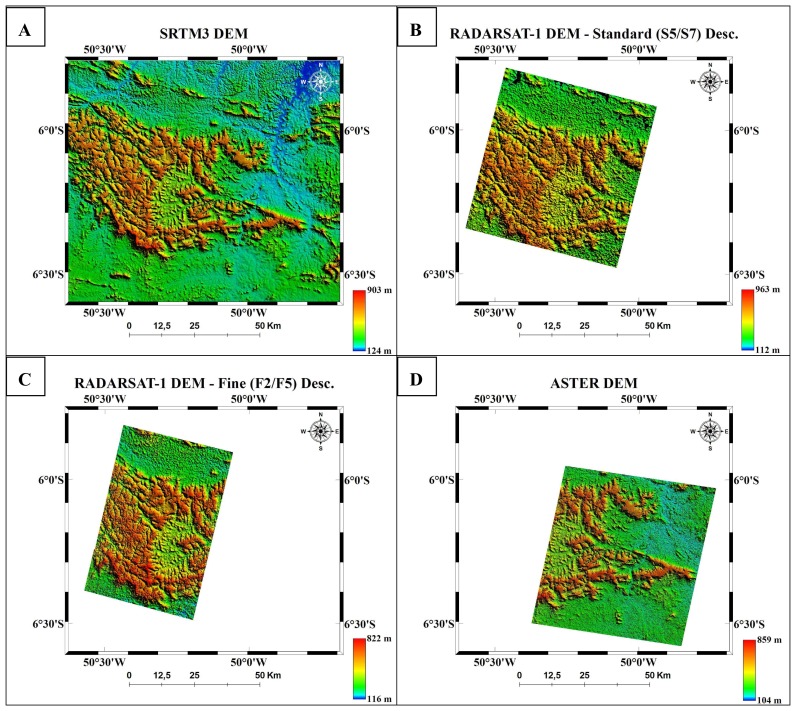
DEMs produced for Carajás with corresponding areas: SRTM3 (A); Standard RADARSAT-1 (B), Fine RADARSAT-1 (C), ASTER (D).

**Figure 3.**
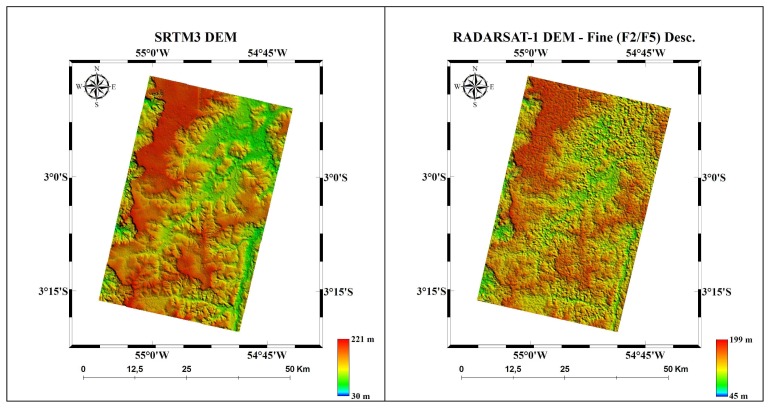
DEMs produced from SRTM3 and RADARSAT-1 Fine for Tapajós area.

Examples of DEMs generated from ASTER, RADARSAT-1 and SRTM3 for Carajás and Tapajós are presented in Figures 2 and 3, respectively. The quantitative results expressed by RMSE, LE90, trend analysis and accuracies are presented in Table 4 and 5, respectively.

The results in the table 4 indicated that using the PEC classification limit (RMSE < 16.66 m) four DEMs can be classified as 1:100,000 scale A Class map. In addition, ASTER presented the best result, followed by SRTM3 (Carajás, Tapajós) and only one representative for RADARSAT-1 (F2/F5 Desc. in Tapajós). With ASTER it was obtained a reduction of 12% of altimetric errors when compared to SRTM3 for the same terrain (Carajás). However, this classification did not consider the presence of systematic errors in the Z direction since SE, Standard Deviation and RMSE were considered equivalents (this only occurs when the mean of the discrepancies is zero). The conclusion to be drawn from the trend analysis (table 5) is that four DEMs presented systematic errors including SRTM3 (t_|x|_ > t_(n−1;5%)_). The more mountainous relief (Carajás), the limited number of GCPs coupled with its distribution, not covering the total area and particularly at the lowest and highest elevations, and the time interval for the scene acquisitions can be considered factors that contributed for the systematic offset (around 20 meters) and large errors found with RADARSAT-1 DEMs in Carajás. In the case of the SRTM3, the observed trends with a positive offset can be explained by the inherent relative characteristic of the DEMs since the phase return in C-band was influenced by the attributes (height, structure and density) of the rainforest [5]. Correction of this vegetation bias has been proposed [15] but this is out of the scope of this paper. Systematic error related to topographic aspect was also considered in the literature [7], which was attributed to the effect of incidence angle of the SAR used to produce SRTM3 DEMs. In addition, the effect of the poor spatial resolution (90 m) could be significant in steep slopes. Finally, the accuracies measured by Chi-square tests indicated that five products were classified as 1:100,000 scale A Class map (*χ*^2^_(A Class)_ < *χ*^2^_(n−1;10%)_).

## 5. Conclusions

The investigation allowed the following conclusions considering the products which were approved under the two approaches (tables 4 and 5): (1) for flat relief (Tapajós), the altimetric accuracy of SRTM3 DEMs and Fine RADARSAT-1 fulfilled the PEC requirements for 1:100,000 A Class Map; (2) for mountainous terrain (Carajás), the altimetry of SRTM3 and ASTER also fulfilled the requirements for 1:100,000 A Class, while Fine and Standard RADARSAT-1 did not; (3) the performance of ASTER was slightly superior to SRTM3. However, it is important to consider the difficulties of acquisition of good stereo-pairs with optical data in the Amazon and the additional cost with GCPs to produce ASTER DEMs. Although only two areas with flat and mountainous terrains had been addressed, the results with SRTM3 justify the choice of this kind of data as a primary elevation source for semi-detailed topographic mapping in the region. The great advantage of SRTM3 is the free access data. However, it is important to mention that for cartographic production up-dated planimetric information is also necessary. Thus, a combination of altimetry derived for SRTM3 and planimetry from high-resolution SAR (PALSAR, TerraSAR-X, RADARSAT-2) or if possible optical data, can be a good alternative to overcome the critical lack of semi-detailed topographic information in the Brazilian Amazon. Finally, if the great expectation of producing stereoscopic DEMs through the new RADARSAT-2 with an accuracy between 5 and 10 m (LE68) using the ultra-fine mode is really confirmed [16], an important market is still open for commercial mapping at 1:50,000 (or better) map scale in large areas of the Amazon.

## Figures and Tables

**Table 1.** Fine RADARSAT-1 stereo-pairs for Carajás.

**Site**	**Carajás**			
Beam	Fine 2 (F2)	Fine 5 (F5)	Fine 2 (F2)	Fine 5 (F5)
Orbit	Descending	Descending	Ascending	Ascending
Date	07/12/2001	15/12/2001	13/02/2002	20/12/2001
Incidence	39° - 42°	45° - 48°	39° - 42°	45° - 48°
Look Azimuth	282°	282°	78°	78°

**Table 2.** Fine RADARSAT-1 stereo-pairs for Tapajós.

**Site**	**Tapajós**	
Beam	Fine 2 (F2)	Fine 5 (F5)
Orbit	Descending	Descending
Date	20/09/2000	27/09/2000
Incidence	39° - 42°	45° - 48°
Look Azimuth	282°	282°

**Table 3.** Standard RADARSAT-1 and ASTER stereo-pairs for Carajás.

**Site**	**Carajás**		
Beam	Standard 5 (S5)	Standard 7 (S7)	ASTER (VNIR-3)
Orbit	Descending	Descending	-
Date	31/05/1996	11/09/1996	16/08/2001
Incidence	36° - 42°	45° - 49°	-
Look Azimuth	282°	282°	-

**Table 4.** Errors for RADARSAT-1, ASTER and SRTM3

**Product**	**Number of GCPs**	**Number of ICPs**	**RMSE (m)**	**LE90 (m)**
F2 Asc. / F5 Asc.^*^	13	20	19.85	32.65
S5 Desc. / S7 Desc.^*^	17	20	25.36	41.71
F2 Desc. / F5 Desc.^*^	20	14	20.11	33.08
F2 Desc. / F5 Desc.^**^	19	24	14.63	24.06
ASTER^*^	20	20	10.57	17.35
SRTM3^*^	-	20	12.10	19.90
SRTM3^**^	-	20	12.79	21.04

* Carajás

** Tapajós

**Table 5.** Trend analysis and accuracy for RADARSAT-1, ASTER and SRTM3.

**Product**	**Mean of the Discrepancies (m)**	**Standard Deviation (m)**	**t_|x|_**	**t_(n−1;5%)_**	**χ^2^(A Class)**	**χ^2^_(n−1; 10%)_**
F2 Asc. / F5 Aasc.^*^	17.23	20.37	3.783	1.729	28.387	27.204
S5 Desc. / S7 Desc.^*^	20.88	26.02	3.589	1.729	46.310	27.204
F2 Desc. / F5 Desc.^*^	− 7.56	18.52	1.527	1.771	16.050	19.812
F2 Desc. / F5 Desc.^**^	4.72	13.81	1.675	1.714	15.793	32.007
ASTER^*^	−0.49	10.56	0.209	1.729	7.623	27.204
SRTM3^*^	10.06	6.31	7.130	1.729	2.726	27.204
SRTM3^**^	9.45	8.34	5.065	1.729	4.763	27.204

* Carajás

** Tapajós
